# Joint Selection of Influential Users and Locations under Target Region in Location-Based Social Networks

**DOI:** 10.3390/s21030709

**Published:** 2021-01-21

**Authors:** Khurshed Ali, Cheng-Te Li, Yi-Shin Chen

**Affiliations:** 1 TIGP-SNHCC, Institute of Information Science, Academia Sinica, Taipei 115, Taiwan; khurshed@iis.sinica.edu.tw; 2 Institute of Systems and Applications, National Tsing Hua University, Hsinchu 300044, Taiwan; yishin@cs.nthu.edu.tw; 3 Institute of Data Science, National Cheng Kung University (NCKU), Tainan 701, Taiwan

**Keywords:** recommendation system, influence maximization, social network analysis, viral marketing, location-based social networks

## Abstract

Influence Maximization problem, selection of a set of users in a social network to maximize the influence spread, has received ample research attention in the social network analysis domain due to its practical applications. Although the problem has been extensively studied, existing works have neglected the location’s popularity and importance along with influential users for product promotion at a particular region in Location-based Social Networks. Real-world marketing companies are more interested in finding suitable locations and influential users in a city to promote their product and attract as many users as possible. In this work, we study the joint selection of influential users and locations within a target region from two complementary perspectives; general and specific location type selection perspectives. The first is to find influential users and locations at a specified region irrespective of location type or category. The second perspective is to recommend locations matching location preference in addition to the target region for product promotion. To address general and specific location recommendations and influential users, we propose heuristic-based methods that effectively find influential users and locations for product promotion. Our experimental results show that it is not always an optimal choice to recommend locations with the highest popularity values, such as ratings, check-ins, and so, which may not be a true indicator of location popularity to be considered for marketing. Our results show that not only influential users are helpful for product promotion, but suitable influential locations can also assist in promoting products in the target region.

## 1. Introduction

Influence Maximization (IM) problem, which finds *k* users from a social network to activate a maximum number of users, is a widely studied research topic [1,2,3,4,5,6]. Domingos and Richardson [7,8] are the first to study the problem as an algorithmic problem and modeled the problem using Markov random fields. The IM problem is formulated as a discrete stochastic optimization problem by Kempe et al. [1] and used two models, i.e., Independent  Cascade (IC) model and Linear Threshold model, to describe the influence propagation over social networks.

In addition, researchers have analyzed the location-based social networks (LBSN) [9,10]. LBSN provides a platform to users to “check-in” and share the location information with their online friends. Considering the popularity of LBSNs, the IM problem has also been studied on location-based social networks (LBSN) [11,12,13,14]. Li et al. [11] have formulated the location-aware influence maximization problem. Given a query region and users’ locations, the problem is to find influential users that could maximize the influence spread in the query region. Bouros et al. [12] have tried to solve a similar problem as proposed in [11] of finding influential users at query region, but he ranked the influential regional users rather than finding a group of users which collectively maximize the influence spread in the query region. Finally, a very recent work by Wang et al. [14] on IM with similar context to our work tries to find regionally influential users considering the distance between seed set and influenced users in a target region. However, none of the previous work has considered a selection of influential users and locations simultaneously for product promotion. Evidently, in a real-world marketing scenario, business companies are as keen on finding suitable venues as selecting a set of key users that could provide maximum profit.

**Figure 1:**
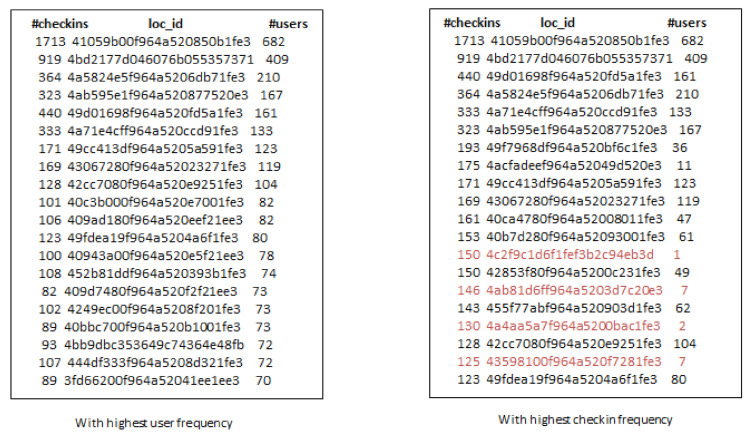
Top-20 locations across San Francisco from Gwalla Dataset.

Due to the popularity of smartphones and location-based social networks (LBSNs), users can check-in at locations or venues and share their check-in information with their friends and family. This free word-of-mouth marketing phenomenon facilitates companies to promote their products by exploring users’ check-in sharing history to attract more users to visit. However, location popularity values, such as check-ins and ratings, are not always available in each location-based social network, except that we are provided only the user social network and set of locations. Thus, it is challenging to find influential users and locations in the target region simultaneously. Further, it is also not sure that the selected locations based on popularity values are always good choices for product promotion. We discuss this issue using the example in Figure 1. In this example, we have selected the top-20 locations across San Francisco from Gwalla dataset based on the number of users and check-in frequency. It can be observed that some locations have the highest check-ins, but these are visited by only few users (marked in red font). So It can be misleading to consider location popularity values only for product promotion. To address this problem, we find influential users and locations which can provide a solid platform for product promotion irrespective of location popularity values.

In short, we answer the following research questions:

1. Is the location check-in frequency always an optimal choice for product promotion in LBSN?
2. How much percent influenced users belong to the query region, and should we consider influenced users out of the region for product promotion?
3. What users and locations to suggest for product promotion matching query region and query topic without considering location popularity information?

To address the aforementioned challenge, we propose two main approaches that select influential users and locations irrespective of location popularity values at the target region. The first approach referred as Region-Aware Influential users and locations selection (IUL), consists of algorithms which select influential users and locations within a target region *R* for product promotion. In-Region (IR Selection) algorithm selects *k* users and *m* locations which are under target region *R*. Region-Free (RF Selection) algorithm considers users who are out of region but connected with users inside target region for finding *k* influential users and *m* influential locations. Greedy-In Region (G-IR Selection) algorithm selects *k*-influential users and *m*-influential locations by considering check-in frequency. It is worth noting that we are not provided the boundary area of the target region except in some LBSNs such as **Foursquare** and so. So we have extracted all the locations which are under a certain distance from the target region. Initially, we find *k* influential users under the target query region and then extract top-*m* locations, visited by *k* influential users, with highest overall users frequency.

The second approach, named topic-aware influential users and locations selection (t-IUL), selects influential users and locations matching the query region and query topic. The algorithms proposed for this approach are similar to the ones proposed in the first approach but with the additional constraint of a specific type of location selection. To sum it all, we make the following contributions.

- We formally define the influential seed and location selection problem over location-based social networks.
- We formally define the topic-aware influential seed and location selection problem over location-based social networks.
- We propose heuristic-based algorithms for influential users and location selection simultaneously for product promotion.

The rest of the paper is organized as follows. Section 2 reviews the related work. Section 3 describes the problem of identifying influential users and locations at a query region in LBSNs. We discuss the proposed frameworks in Section 4. Section 5 show our experimental results, followed by discussion and conclusion in Section 6.

## 2. Literature Review

### 2.1. Influence Maximization in Social Network

The influence maximization (IM) problem has been extensively studied in literature [1,2,3,4,5,7,8,11,15]. The IM problem was proposed by Domingos et al. in their seminal work [7]. The authors proposed probabilistic methods to address the IM problem. Kempe et al. [1] utilized two models, Independent cascade (IC) and Linear threshold (LT), to solve the IM problem. Futher, he proved the monotonic and the submodular property of the influence spread function and the problem’s hardness (i.e. NP-hard). As the problem is NP-hard, the authors proposed the greedy algorithms with the approximation ratio of $\left(1-\frac{1}{e}\right)$. Further, research is carried out to improve the efficiency and scalability of the greedy algorithm while maintaining the approximation ratio. Leskovec et al. [15] proposed a lazy forward approach which achieves 700 times better efficiency performance than the algorithm proposed in [1]. Chen et al. [2] proved that it is #P-hard to compute the influence spread. Thus, many algorithms rely on heuristic strategies to enhance performance. Chen et al. [2] utilized the degree discount heuristic to compute the influence spread for influence maximization problem. Recently, a near-linear time approach is proposed by Borgs et al. [4], which utilizes the Monte-Carlo Simulations to compute the influence spread. Further, Ohsaka et al. [5] proposed a pruning technique for reducing Monte-Carlo simulations time complexity to achieve better results with theoretical guarantees.

### 2.2. Influence Maximization in Geo-Social Network

With the increase in the use of location-enabled devices, the location factor carries an important role in social network analysis. Zhu et al. [13] consider a geo-social network where each user is associated with multiple check-ins. The authors proposed algorithms to compute the propagation probability distribution based on user check-in history in a promoted location. The works mostly related to our work are [11,12,14]. Li et al. [11] selected a seed set which globally maximizes the influence spread in a query region. Bouros et al. [12] used the Dijkstra algorithm to compute the regional influence of all users at a specific region and rank the influential users accordingly. Wang et al. [14], have considered a distance between seed set and promoted location for assigning edge weight. They have assigned different weights to users based on the distance between a user and the promoted location.

All of the aforementioned works have considered the location popularity and tried to find the seed set, which can globally maximize the influence spread. Our work mostly resembles to the work proposed by Bouros et al. [12], but we recommend influential locations along with an influential seed set for product promotion at a target region in location-based social networks.

### 2.3. Location Recommendation in LBSNs

Researchers have investigated the location-based social networks for analysis as well [9,10,16]. Authors in [9] analyzed the LBSN, its properties, and possible issues related to it. While, Ahmed et al. [16] discussed the importance of different centrality measures for identifying a node in geo-based social networks. Besides, Venue or location recommendation matching user query in LBSN has been well studied in literature [17,18,19,20,21,22]. Ye et al. [21] used collaborative filtering recommendation system by developing *friend-based collaborative filtering* system to recommend locations to users. Levandoski et al. [18] used the user ratings to locations for venue recommendation. Yuan et al. [17] proposed a *time-aware POI recommendation problem*, which suggests a list of venues for a user to visit at a given time. A recent work by Zhang et al. [23] recommends locations based on users preferences learning from the community-contributed data. The work done by Chang et al. [23] is similar to our *t-IUL* problem, but we recommend venues to merchants which satisfy merchant’s query topic and target region by considering influential users in mind.

## 3. Problem Formulation

A LBSN $\langle G,L\rangle$ consists of a social network G=(U,E), where *U* is the set of users, *E* is the set of edges and the set *L* represents the set of locations with users check-in history as $\left\{(u,l_{1},t)\right\}$, where $\left(u,l_{1},t\right)$ represents a check-in record of user *u* at location $l_{1}$ at time *t*, and $l_{1}\in L$. A location $l_{1}$ consists of latitude, longitude and location id. For *t-IUL* problem, we assume that each location has category type which defines the type of location.

**Definition** **1.** *Influential Users: Influential users are a set of key users selected by companies that can propagate companies product information to a large number of users in a social network.*

**Definition** **2.** *Influential Locations: Influential locations are locations that help companies spread their product information to a large volume of users even without investing in seed users set.*

Now, we formally define the joint selection of influential users and locations problem (IUL) as following.

**Definition** **3.** *IUL Problem: Given a LBSN $\langle G,L\rangle$, a query region R, constants k and m, the IUL problem is to select a set of users S, where S∈U,|S|=k, and set of locations V, where V∈L,|V|=m, which both can help promote products at target region R and attract as many users as possible to adopt products.*

We illustrate the importance of the joint selection of influential users and locations in the following example.

**Figure 2:**
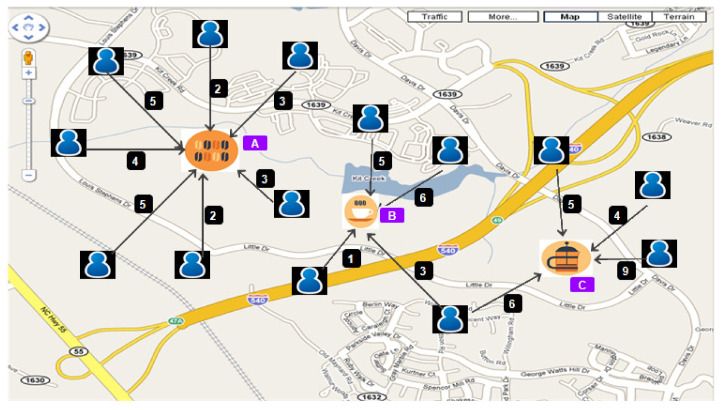
Example Figure.

**Example** **1.** *Figure 2 shows locations with users check-in frequency. We suppose all locations lie under a target query region. Under this query region, top-2 locations (m=2) would be locations A and location C having the most users and highest check-ins, respectively. Although top-2 locations are locations A and C, in fact, we do not know the actual visited users and check-ins at the target region. So, it is not easy to find influential users and locations under a query region for product  promotion.*

Next, we define the Topic-Aware selection of influential users and locations (*t-IUL*) problem as follows.

**Definition** **4.** *t-IUL Problem: Given a LBSN $\langle G,L\rangle$, a target region R, a constant m and a location category C, the t-IUL Problem is to select a set of seeds S, where S∈U,|S|=k, and set of locations V, where V∈L,|V|=m, matching a query category C, which can help companies to promote product at target region R and spread their product information to large number of users in a target region.*

## 4. Methodology

Section 4.1 discusses the approaches for solving *IUL* problem followed by strategies for solving *t-IUL* problem in Section 4.2.

### 4.1. IUL Approaches

Given a LBSN $\langle G,L\rangle$, a target region *R*, constants *k* and *m*, the problem is to select *k* influential users and *m* influential locations that can help to promote products. Initially, we extract all the locations which are within target query region *R* followed by social sub-graph construction of users who have checked-in at extracted locations. Then influential *k*-seeds are found by using degree discount proposed by Chen et al. [2]. It should be noted that we can find influential *k*-seeds using other approaches too, such as proposed by Ohsaka et al. [5], and Borgs et al. [4]. Finally, we select all locations visited by *k*-seeds and select top-*m* locations from these locations based on highest number of users visiting those locations.

We propose three heuristic-based methods, In-Region Selection *IR Selection*, Region-Free Selection *RF Selection* and Greedy In-Region Selection *G-IR*, to address the *IUL* problem.

**IR Selection**. In this method, we consider all those users who have checked-in at least once within a query region boundary for influential seeds as well as the targeted users who can be influenced by product promotion. This approach is suitable when the marketing company wants to target the users who have frequently visited the places within a query region boundary and have a social connection with each other.

The Algorithm 1 selects the influential users and locations within a target query region *R* using the IR Selection approach.
**Algorithm 1** IR influential users and locations selection.     **Input:** LBSN: $\langle G,L\rangle$; Query: (R,k,m)     **Output:** *S*-seeds, *V*-locations 11:S←∅,V←∅2:θ← 150 miles                    ▹The boundary area for target region *R*3:$L^{\prime }\leftarrow \emptyset$                               ▹Location set4:I← 10,000                        ▹Number of Simulations5:**for all** locations *l* ∈ *L* **do**6:    **if** distance(*l*, R) ≤θ **then**7:        $L^{\prime }=L^{\prime }\cup \left\{l\right\}$8:Extract Users checked in at $L^{\prime }$9:$G^{\prime }=\left(U^{\prime },E^{\prime }\right)|\left\{U^{\prime }\subseteq U\wedge U^{\prime }\exists L^{\prime }\right\}$  ▹Subgraph of users who checked in at locations lying under target region10:$S\leftarrow DegreeDiscount(G^{\prime },k,I)$11:$L^{\prime \prime }\subseteq L^{\prime }|\left\{S\exists L^{\prime \prime }\right\}$               ▹extract locations visited by top S seed users12:**for**i←1:m**do**13:    **for all** locations *l* $\in L^{\prime \prime }$ **do**14:        $L_{UF}=ComputeLocationUserFreq\left(l\right)$15:    $V=V\cup \{{arg max}_{l\in L_{UF}\setminus V}\left\{L_{UF}\right\}\}$16:    $L^{\prime \prime }=L^{\prime \prime }-V$17:Return S,V

In this Algorithm 1, we consider users within a target query region *R*. Line 2 computes the boundary area of a target region *R*. Here, we assume the boundary area of each target city as 150 miles. It means we extract all locations which lie under 150 miles of a target region/city (i.e., New York). $L^{\prime }$ and *I* represent the locations within target region and number of simulations to run for influence at line 3 and 5 respectively. We extract all the locations which lie under target region boundary from lines 5 to 7. After getting all the locations within a target region, a social sub-graph, $G^{\prime }$, is constructed of users who checked-in at those locations, i.e., line 8 selects users who checked in at locations $L^{\prime }$ which lie under a target query region. We extract top-k seeds at line 9 using the degree discount method. Line 10 of this algorithm extracts the locations, $L^{\prime \prime }$, visited by top-*k* influential seeds. From line 14 to 15, we compute the location’s user visiting frequency, $L^{\prime \prime }$, and each location is stored along with the number of users (not only top-k seeds but all users) who checked-in at these locations. Top-*m* locations are selected by extracting locations with highest user frequency from $L^{\prime \prime }$ at line 17 to 19.

**RF Selection**: In the region-free selection approach, users who have checked-in at least once at query region *R* boundary are selected along with the inclusion of their children who can be within target region or out of target region boundary. As we are not provided the user’s home location, so, we consider users having social links with users inside the target region boundary. This approach is beneficial when we do not know the actual home location of users but can target them if they have any social ties with users inside the target region.

The Algorithm 2 discusses the selection of influential users and locations using the RF selection approach. In this algorithm, line 1 extracts all those users who have checked-in at least once within a target query region, *R*, boundary first. Next, we add the child nodes of users who were selected earlier (at line 1) from lines 3 to 6. Further, we select the *k*-influential users and *m* influential locations from line 7 to line 13 in the same way as discussed in Algorithm 1.
**Algorithm 2** RF Selection of influential users and locations.     **Input:** LBSN: $\langle G,L\rangle$; Query: (R,k,m)     **Output:** *S*-seeds, *V*-locations 1     Initialization and locations extraction, $L^{\prime }$, are same as in Algorithm 1.2:Extract Users checked in at $L^{\prime }$$G^{\prime }=\left(U^{\prime },E^{\prime }\right)|\left\{U^{\prime }\subseteq U\wedge U^{\prime }\exists L^{\prime }\right\}$        ▹Subgraph of users who checked in at locations lying under target region4:$G^{\prime \prime }\leftarrow G^{\prime }$**for all** users *u* $\in U^{\prime }$ **do**6:    **for** each neighbor *v* of *u* **do**        **if** (*v* not in $G^{\prime \prime }$) **then**8:           $G^{\prime \prime }=G^{\prime \prime }\cup v$$S\leftarrow DegreeDiscount(G^{\prime \prime },k,I)$10:$L^{\prime \prime }\subseteq L^{\prime }|\left\{S\exists L^{\prime \prime }\right\}$            ▹extract locations visited by top S seed users**for**i←1:m**do**12:    **for all** locations *l* $\in L^{\prime \prime }$ **do**        $L_{UF}=ComputeLocationUserFreq\left(l\right)$14:    $V=V\cup \{{arg max}_{l\in L_{UF}\setminus V}\left\{L_{UF}\right\}\}$    $L^{\prime \prime }=L^{\prime \prime }-V$16:Return S,V

**G-IR Selection**. The Greedy in-region selection approach selects top-*k* influential users and *m*-locations with highest number of check-ins. It is assumed that locations and users with the highest number of check-ins are suitable for product promotion in the target region and can spread product information to many users in a region.

The Algorithm 3 discusses the greedy approach for finding top-*k* influential users and *m*-influential locations. Line 1 extracts the users who have checked-in at target query region *R*. From lines 2 to 6, we select *k*-influential users considering the user’s highest number of check-ins. Next, we select top *m*-influential locations with the most number of visits, from line 7 to 13.

Each proposed heuristic-based methods carries pros and cons with it. For instance, the IR-Selection approach focuses only on users who are within the target region boundary but lacks users consideration who are indirectly inside the target region boundary. The RF-Selection approach considers both users, i.e., users directly inside region boundary and indirectly within region boundary, for product promotion.
**Algorithm 3** G-IR Selection of influential users and locations.     **Input:** LBSN: $\langle G,L\rangle$; Query: (R,k,m)     **Output:** *S*-seeds, *V*-locations 1     Initialization and locations extraction, $L^{\prime }$, are same as in Algorithm 1.     Extract Users checked in at $L^{\prime }$3:$G^{\prime }=\left(U^{\prime },E^{\prime }\right)|\left\{U^{\prime }\subseteq U\wedge U^{\prime }\exists L^{\prime }\right\}$     ▹ Subgraph of users who checked in at location lying under target region**for**i←1:k**do**    **for all** users *u* $\in U^{\prime }$ **do**6:        $U_{CF}^{\prime }=ComputeUserCheckinFreq\left(u\right)$    $S=S\cup \{{arg max}_{u\in U_{CF}^{\prime }\setminus U^{\prime }}\left\{U_{UF}^{\prime }\right\}\}$    $U^{\prime }=U^{\prime }-S$9:**for**i←1:m**do**    **for all** locations *l* $\in L^{\prime }$ **do**        $L_{UF}=ComputeLocationUserFreq\left(l\right)$12:    $V=V\cup \{{arg max}_{l\in L_{UF}\setminus V}\left\{L_{UF}\right\}\}$    $L^{\prime }=L^{\prime }-V$Return S,V

Further, the effectiveness of each approach, i.e., In-Region Selection (IR Selection), Region-Free Selection (RF Selection), and Greedy In-Region Selection (G-IR), is discussed in Section 5.

### 4.2. t-IUL Approaches

*t-IUL* problem is to select influential users and locations attarget query region matching a specific query topic.

We propose two heuristic-based approaches to solve *t-IUL* problem; topic-aware Region-Free Selection (t-RF selection) and topic-aware Greedy In-Region Selection (t-GIR selection).

**t-RF Selection**. Topic-Aware Region free selection approach is similar to the **RF-Selection** approach of *IUL* problem with one more constraint in location selection. Here, we consider locations lying within a target query region boundary and matching the query topic that can be the merchant’s own interest or preference for product promotion.

The Algorithm 4 illustrates our topic-aware region free selection approach. We compare the location type/category with a given query type. We have designed this algorithm considering *Foursquare* dataset, which have location city and category information. It can be extended to any other LBSNs dataset, provided that every location has a category or topic field. Lines 2 to 5 of this algorithm extracts those locations which match the target query region and query topic. The remaining part of the algorithm is the same as the RF-Selection algorithm and discussed earlier in *IUL approach*.
**Algorithm 4** t-RF Selection of influential users and locations.     **Input:** LBSN: $\langle G,L\rangle$; Query: (R,k,m,q)     **Output:** *S*-seeds, *V*-locations 1     I← 10,000                 ▹ Number of Simulations     **for all** locations *l* ∈ *L* **do**          **if** (*l*.city ==R) and (l.category == q) **then**4:        $L^{\prime }=L^{\prime }\cup \left\{l\right\}$$G^{\prime }=\left(U^{\prime },E^{\prime }\right)|\left\{U^{\prime }\subseteq U\wedge U^{\prime }\exists L^{\prime }\right\}$    ▹ Subgraph of users who checked in at location lying under target region$G^{\prime \prime }\leftarrow G^{\prime }$**for all** users *u* $\in U^{\prime }$ **do**8:    **for** each neighbor *v* of *u* **do**        **if** (*v* not in $G^{\prime \prime }$) **then**           $G^{\prime \prime }=G^{\prime \prime }\cup \left(v\right)$$S\leftarrow DegreeDiscount(G^{\prime \prime },k,I)$12:$L^{\prime \prime }\subseteq L^{\prime }|\left\{S\exists L^{\prime \prime }\right\}$**for**i←1:m**do**    **for all** locations *l* $\in L^{\prime \prime }$ **do**        $L_{UF}=ComputeLocationUserFreq\left(l\right)$16:    $V=V\cup \{{arg max}_{l\in L_{UF}\setminus V}\left\{L_{UF}\right\}\}$    $L^{\prime }=L^{\prime }-V$Return S,V

**t-GIR Selection**. Topic-Aware Greedy In-Region approach resembles the **G-IR Selection** approach of *IUL* problem with additional constraint in location selection. Here, we consider locations belonging to the target query region and matching the merchant’s preferred query topic for product promotion.

The Algorithm 5 presents the greedy approach for selecting influential users and locations matching user query topic. It is worth noting that these both Algorithms 4 and 5, can easily be used to find *m*-influential locations to recommend to users for visiting (from consumer’s perspective).
**Algorithm 5** t-GIR Selection of influential users and locations.     **Input:** LBSN: $\langle G,L\rangle$; Query: (R,k,m,q)     **Output:** *S*-seeds, *V*-locations 11:Initialization and locations extraction, $L^{\prime }$, are same as in Algorithm 4.2:$G^{\prime }=\left(U^{\prime },E^{\prime }\right)|\left\{U^{\prime }\subseteq U\wedge U^{\prime }\exists L^{\prime }\right\}$3:**for**i←1:k**do**4:    **for all** users *u* $\in U^{\prime }$ **do**5:        $U_{CF}^{\prime }=ComputeUserCheckinFreq\left(u\right)$6:    $S=S\cup \{{arg max}_{u\in U_{CF}^{\prime }\setminus U^{\prime }}\left\{U_{UF}^{\prime }\right\}\}$7:    $U^{\prime }=U^{\prime }-S$8:**for**i←1:m**do**9:    **for all** locations *l* $\in L^{\prime }$ **do**10:        $L_{UF}=ComputeLocationUserFreq\left(l\right)$11:    $V=V\cup \{{arg max}_{l\in L_{UF}\setminus V}\left\{L_{UF}\right\}\}$12:    $L^{\prime }=L^{\prime }-V$13:Return S,V

### 4.3. Time Complexity

**Time Complexity**: Let *L* denotes the total number of locations in LBSN, $L^{\prime }$ represents number of locations in target region, $L^{\prime \prime }$ number of locations visited by influential seeds. And *n* represents the total number of users, $n^{\prime }$- number of users checked-in at target region, *m*-number of edges.

Algorithms 1 and 2 use the degree discount [2] heuristic-based method. As mentioned by Chen et al. [2], the running time of it is $O(k\log n^{\prime }+m)$. So the running time of Algorithm 1 is $O(L+nL^{\prime }+k\log n+m)$ and the running time of Algorithm 2 is $O(L+nL^{\prime }+nn^{\prime }+k\log n+m)$. Though the Algorithm 1 is faster than the Algorithm 2 but it lacks user selection who have directly social connection with users inside target region and could be useful for marketing the company products.

The running time of Algorithm 3 is $O(L+nL^{\prime }+kn^{\prime }+mL^{\prime })$. The greedy In-Region selection approach is faster than the other two approaches. However, it does not provide users and locations which are beneficial for product promotion as discussed in Section 5.

The time complexity of Algorithms 4 and 5 is the same as the running time complexity of Algorithm 2 and 3, respectively.

Further, we discuss the effectiveness of our proposed algorithms in results Section 5.

## 5. Experiments

We conducted experiments of our proposed algorithms on three real-world location-based social networks, as discussed in Table 1. The objective is to find which algorithms can provide better and effective results for product promotion under certain constraints, such as within target region *R* and location type *q*. Besides, we analyze the recommended users and locations for product promotions about how and why these results would be helpful for product promotion in a target region.

### 5.1. Experimental Setup

**Datasets**. We used three real world datasets **Gowolla**, **Brighkite**, **Foursquare**. Gowalla and Brighkite data sets are publicly available at stanford website [24]. While the Foursquare date set (http://net.pku.edu.cn/daim/yinhongzhi/index.html), used by Wang et al. in [25], was downloaded from his website (publicly made available for download). The three datasets are directed graphs and details of each data set is discussed in Table 1.

**Comparison Methods**. We compare our heuristic-based algorithms (Algorithms 1, 2 and 4 ) with greedy approaches (Algorithms 3 and 5). For the heuristic methods, we used the degree discount approach [2] to find the seed set and compute the influence spread. Finding the influential users and their influence spread can easily be done by other state-of-the-art approaches, such as [4,5,6].

**Target Query Region**. We have selected the big modern cities of the U.S., such as New York, Chicago, San Francisco, Washington, Seattle, Las Angeles, as our target query region and computed the influential users and locations selection within a specified target region. For each city, we extracted its geo-coordinates, i.e., latitude and longitude, and then selected all those check-in venues which lie under the target region. Locations within the target region are extracted using Vincenty distance formula. Since the Foursquare dataset contains city information, so we extracted all locations matching the query region.

**Propagation Probability**. We randomly selected each edge’s propagation probability from {0.1,0.01,0.5,0.001} as mentioned in [3].

**Parameters**. We measure the effectiveness of the proposed approaches for reporting region-aware influence spread, comparing selected influential users and locations. The seed set size *k* varies from 10 to 50 and set m-locations as 20.

The influence spread of heuristic approaches is obtained by running 10,000 simulations for each set and took the average of the influence spread that matches the settings in [3]. All the proposed approaches are implemented in Python. Experiments are conducted on a PC with Intel Core(TM) i7 3.6 GHz and 8 GB memory using Windows 10.

### 5.2. Experimental Results

We evaluate the effectiveness of heuristic-based algorithms with greedy-based algorithms proposed in *IUL* and *t-IUL* approaches by varying seed set size *k*. We discuss the experimental results of *IUL* and *t-IUL* approaches in Section 5.2.1 and Section 5.2.2 respectively.

#### 5.2.1. IUL Approaches Result Summary

First, we discuss the influence spread result achieved by IR, RF, and G-IR selection algorithms. As all the algorithms showed similar behavior on the Brightkite dataset as they performed on the Gowalla dataset, so we have excluded the Brightkite analysis result summary here.

**Figure 3:**
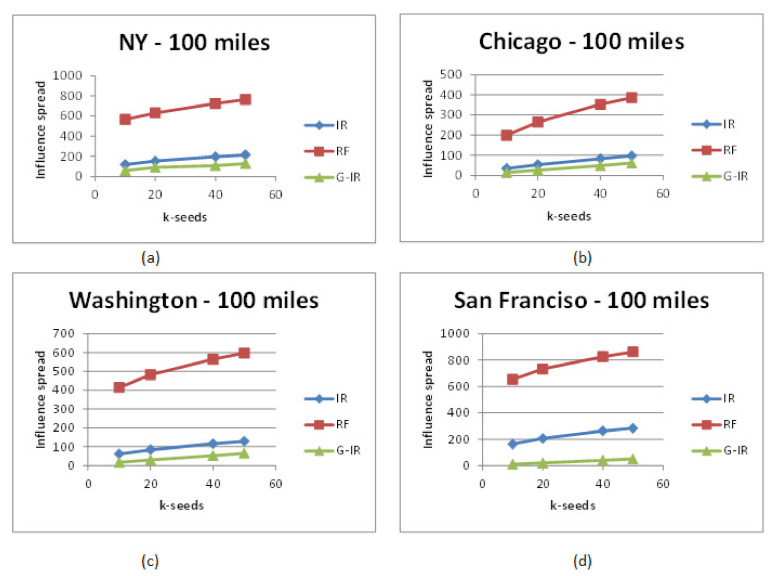
Influence spread on various regions on Gwalla dataset.

Figure 3 shows the influence spread within or out of target cities where locations lie under 150 miles of the target city on the Gowalla dataset. It can be seen that there is a huge difference in influence spread within and out of the target city achieved by RF, IR, and G-IR algorithms. Like in Figure 3a, when k = 20, the number of influenced users achieved by G-IR, IR, and RF algorithms are around 100, 180, and 650, respectively. There is a significant percentage difference in influence spread within and out of the target region. Similar difference is observed in other target cities as shown in Figure 3b–d. We conducted the same experiment on other cities of U.S., such as Seattle, California, Las Angles, and Los Vegas, and found the same influence spread difference in these target cities as well.

**Figure 4:**
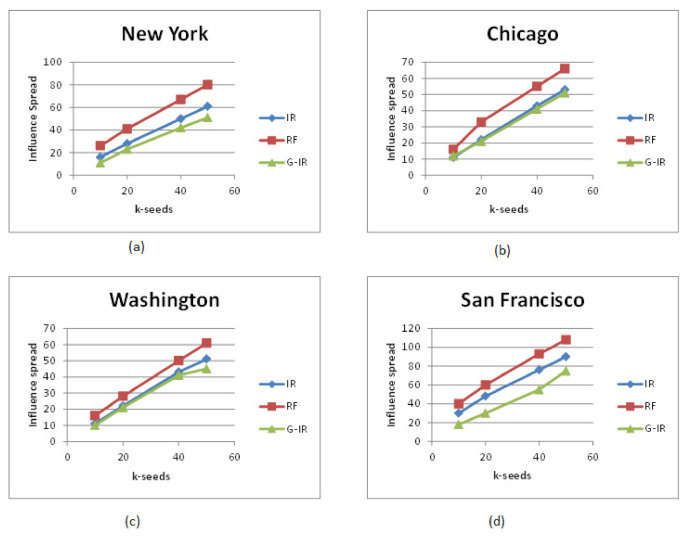
Influence spread on various regions on Foursquare dataset.

Figure 4 shows the results of influence spread of big cities of U.S. on Foursquare dataset. Since we do not have a considerable number of users and location check-in information of the Foursquare dataset, so it looks like that there is not much significant difference in influence spread achieved by all these algorithms. But we can observe the influence spread difference once the provided social network is large and consists of a vast amount of locations with check-in information.

It is evident from Figure 3 and Figure 4 that the RF Selection algorithm achieved better results than IR Selection and G-IR Selection algorithms. IR and G-IR algorithms achieved similar influence spread in most of the cities on Foursquare datasets except in some cities where IR-algorithm performed better than G-IR.

From these experimental results, we believe that the top k-influential seed set who are local to the target city and have friends across the region can influence users across the target city and motivate them to visit famous locations under target cities.

Next, we discuss the effectiveness of a joint selection of influential users and locations selected by IR, RF, and G-IR algorithms.

**Figure 5:**
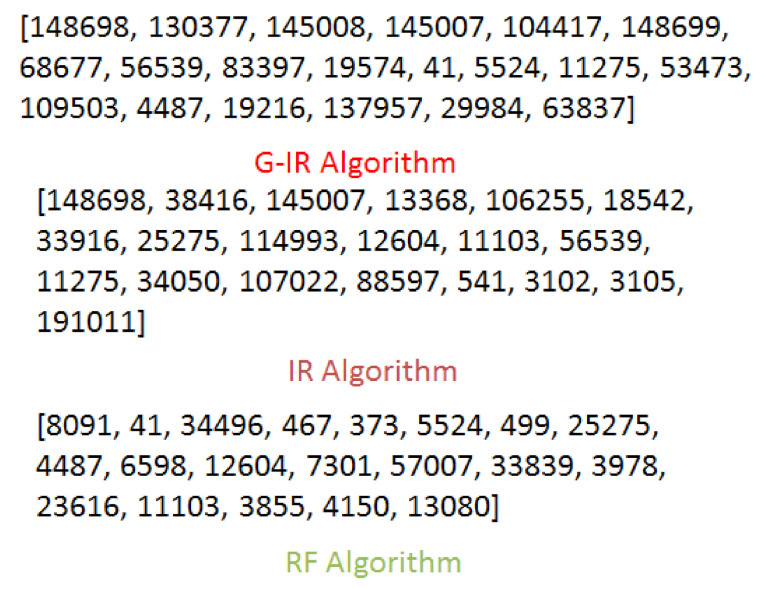
Top-20 influential Users across San Francisco on Gwalla dataset.

Figure 5 shows the top-*k* (*k* = 20) influential users selected by G-IR, IR and FR algorithms respectively. It is observed that each algorithm produce a different *k*-influential seed set, so it is better to select the seed set which could maximize the product promotion. Further, we discuss the influential locations selected by each algorithm.

**Figure 6:**
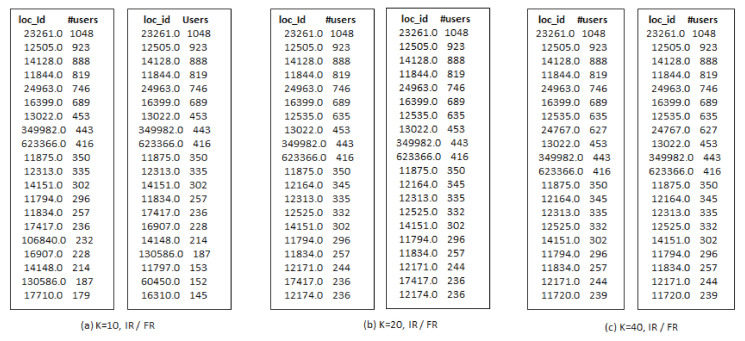
Top-20 influential locations selected by IR and RF algorithms across New York.

**Figure 7:**
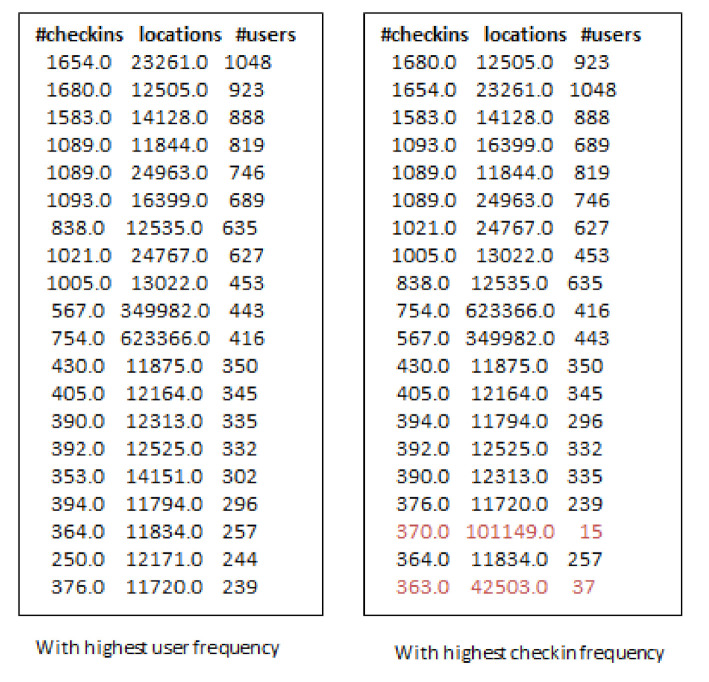
Top-20 influential locations across New York using G-IR algorithm.

Figure 6 shows the top-m (m = 20), influential locations selected by IR and FR selection algorithms with varying k-seeds. Figure 7 shows the same number of locations selected by the G-IR selection algorithm. It can be observed that when we increase the number of k-seeds, our proposed approach achieves better and similar influential locations as selected by a greedy approach with the highest users. However, the greedy approach (G-IR), which considers only check-in frequency distribution, yields poor results in influential locations selection. Locations selected by G-IR highlighted in red color shows the venues with less number of user visiting frequency than heuristic-based approaches.

**Figure 8:**
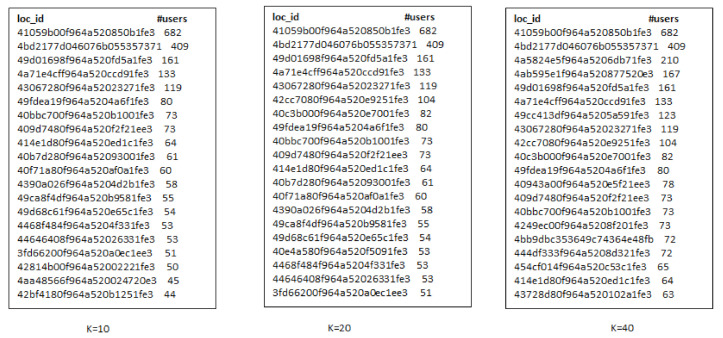
Top-20 influential locations across San Francisco using RF-algorithm on Foursquare.

**Figure 9:**
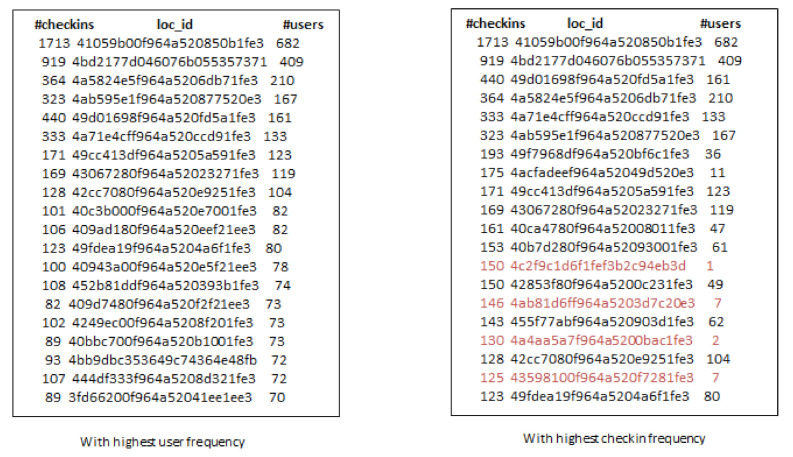
Top-20 influential locations across San Francisco using GIR algorithm.

Further, we conducted the same experiment for selecting influential locations on Foursquare and Gwalla datasets as well. Figure 8 and Figure 9 show the results obtained by RF and GR-Selection algorithms on Foursquare dataset. Locations selected by G-IR highlighted in red color shows the venues with less number of users visiting frequency than our approach.

#### 5.2.2. t-IUL Approaches Result Summary

We conducted the experiments for *t-IUL* problem on *Foursquare* dataset only because it have location category information while *Gowolla* and *Brighkite* does not have any location category information. We selected the target region, *R*, as *Log Angeles* and category topic, *q*, as *food*. We have considered "food" as a location category type as users are found to share favorite food locations with their friends. Here, we discuss the influential locations selected by t-RF and t-GIR algorithms and see the results difference produced by both algorithms. Influential seeds, k, selected by t-RF and t-GIR algorithms are almost the same for the Foursquare dataset, so we have not shown that result in this paper.

**Figure 10:**
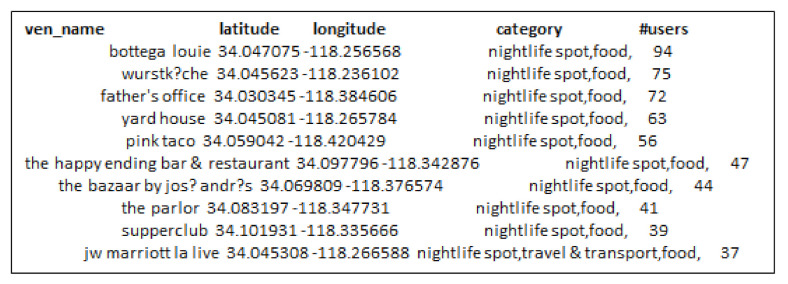
Top-10 influential locations in Log Angles using t-RF algorithm.

**Figure 11:**
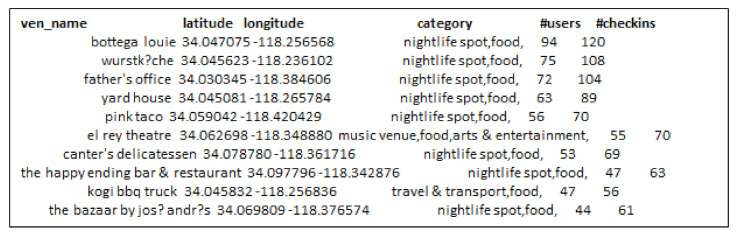
Top-10 influential locations in Log Angles using t-GIR algorithm.

**Figure 12:**
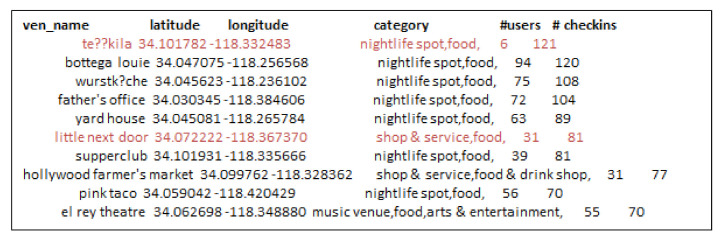
Top-10 influential locations in Log Angles using t-GIR algorithm considering check-ins.

Figure 10 shows the top-10 influential locations selected by the t-RF algorithm. We found around 5.3 k check-ins matching query category and target city. Besides, we set k-seeds as 50 because there are not many users in *Foursquare* dataset. Influential users visit the 95 unique venues. Further, Figure 11 and Figure 12 show the venues selected by *t-GIR* Selection algorithm considering highest users with check-in frequency. The venues highlighted in red color shows the drawback of *t-GIR* algorithm because it returns locations with the highest check-ins, but only a few users have visited those places

### 5.3. Discussion

The experimental results provides the following analysis.

- There is a significant percentage difference in influence spread within and out of the target region.
- RF-algorithms achieved better performance as they considered users having connections across the target city for promotion. The top k-influential seed set who are local to the target city and have friends across the region can influence users to visit famous locations under target city.
- Third, the locations selected by considering check-in information only are not always an optimal choice for production promotion. Since, few users can have checked-in location hundreds or thousands times but it may not denote its real popularity.
- When we increase the number of k-seeds, our proposed approach achieves better influential locations as selected by a greedy approach with the highest users. However, the greedy approach (G-IR), which considers only check-in frequency distribution, yields poor results in influential locations selection.

## 6. Conclusions

In this paper, we have studied the problem of finding influential users and locations jointly at the target query region for product promotion. Further, we studied the selection of influential users and locations matching the specific location category. As location popularity values, such as the number of check-ins, are not always available, so we have proposed heuristic-based methods that can find influential users and locations in the target region. Experimental results showed that our heuristic-based methods achieved better performance than the greedy-based methods. Further, our proposed algorithms can easily be extended by adding more constraints such as recommending top *M* locations, by considering the distance between each location, i.e., we can suggest influential locations for product promotion which should be distant apart from each other. In future work, we would like to find a co-relation between *k*-influential users and *m*-influential locations under target region in location-based social networks.

## Figures and Tables

**Table 1:** Datasets.

Datasets	#Vertices	#Edges	#Check-ins
Gowalla	196,591	950,327	6.4 M
Brighkite	58,228	214,078	4.49 M
Foursquare	4163	32,512	483,813

## Data Availability

The data presented in this study are available on request from the corresponding author.

## Abbreviations

The following abbreviations are used in this manuscript:

MDPI	Multidisciplinary Digital Publishing Institute
IM	Influence Maximization
LBSNs	Location-based Social Networks
IUL	Influential Users and Locations
t-IUL	Topic-aware Influential Users and Locations
IR	In-Region
RF	Region-Free
G-IR	Greedy In-Region
IC	Independent cascade
LT	Linear threshold
t-RF	Topic-aware Region-Free
